# Warwick Hip Trauma Study: a randomised clinical trial comparing interventions to improve outcomes in internally fixed intracapsular fractures of the proximal femur. Protocol for The WHiT Study

**DOI:** 10.1186/1471-2474-11-184

**Published:** 2010-08-17

**Authors:** Xavier Luke Griffin, Nick Parsons, Juul Achten, Matthew L Costa

**Affiliations:** 1 Clinical Sciences Research Institute, University of Warwick Medical School, Clifford Bridge Road, Coventry, CV2 2DX, UK

## Abstract

**Background:**

Controversy exists regarding the optimal treatment for patients with displaced intracapsular fractures of the proximal femur. The recognised treatment alternatives are arthroplasty and internal fixation. The principal criticism of internal fixation is the high rate of non-union; up to 30% of patients will have a failure of the fixation leading to revision surgery. We believe that improved fracture healing may lead to a decreased rate of failure of fixation. We therefore propose to investigate strategies to both accelerate fracture healing and improve fixation that may significantly improve outcomes after internal fixation of intracapsular femoral fractures. We aim to test the clinical effectiveness of the osteoinductive agent platelet rich plasma and conduct a pilot study of a novel fixed-angle fixation system.

**Design:**

We have planned a three arm, single centre, standard-of-care controlled, double blinded, pragmatic, randomised clinical trial. The trial will include a standard two-way comparison between platelet-rich plasma and standard-of-care fixation versus standard-of-care fixation alone. In addition there will be a subsidiary pilot arm testing a fixed-angle screw and plate fixation system.

**Trial Registration:**

Current Controlled Trials ISRCTN49197425

## Background

### Epidemiology

Proximal femoral fractures are one of the greatest challenges facing the medical community. In 1990, a global incidence of 1.31 million was reported and was associated with 740,000 deaths [1]. Proximal femoral fractures constitute a heavy socioeconomic burden worldwide. The cost of this clinical problem is estimated at 1.75 million disability adjusted life years lost, 1.4% of the total healthcare burden in established market economies [1].

### Existing knowledge

Proximal femoral fractures can be subdivided into intra and extracapsular fractures. Approximately half of all proximal femoral fractures are intracapsular. These fractures are at risk of healing complications as the blood supply to the femoral head may be compromised by the fracture. There are two operative strategies in the management of intracapsular fractures of the proximal femur: internal fixation and hip arthroplasty.

Arthroplasty surgery eliminates the risk of fixation failure as the femoral head is replaced. However, it is a major operation with very significant complications of its own including infection, dislocation and periprosthetic fracture. The most common form of arthroplasty in this group of patients is hemiarthroplasty, where the head of the femur is replaced but the acetabulum is left intact, but this procedure is associated with an approximately 20% risk of late acetabular wear leading to arthritic changes and the potential need for further surgery [2]. Internal fixation has the key advantage of preserving the patients' own bone and cartilage. It is also a quicker operation requiring a much smaller wound. The principal complication of internal fixation is non-union which is related to the tenuous blood supply to the femoral head. However, the rate of non-union and fixation failure has been reported at up to 33%, [3] leading to re-operation in 90% of these patients. Consequently, the best treatment of these fractures remains controversial. A recent Cochrane review [4] has confirmed that the evidence suggests that there is no clinical benefit of one treatment over the other.

In order for any fracture to heal successfully there must be both a good biological environment and adequate fracture fixation. When a fracture heals there is a balance between the time required to achieve union and the time over which the fixation maintains fracture position. Therefore, the failure of a fracture to heal may be due to an inadequate biological environment (leading to a long healing time) or an inadequate fixation system (leading to a short period of effective fixation). Interventions to improve fracture healing are targeted at one of these two broad areas. In patients with intracapsular fractures of the proximal femur interventions to improve fracture healing may reduce the rate of fixation failure and therefore the requirement for major arthroplasty surgery.

### Aim of the trial

The aim of this trial is to investigate the clinical effectiveness of novel surgical interventions to improve clinical outcomes following fracture of the proximal femur. Currently, there are two new techniques available which have shown promising early results for the treatment of acute fractures: firstly, platelet-rich plasma (PRP) which is an autologous source of growth factors derived from a patient's whole blood; secondly, novel fixed-angle screw and plate systems which are available following developments in the field of fragility fracture fixation. Early results of both these interventions are promising but there is no Level I clinical data [5,6].

### Hypothesis

We propose to test the hypotheses that: PRP leads to a reduced incidence of failure of fixation in patients with intracapsular fractures of the proximal femur.

We propose to explore the size of any treatment effect due to a novel fixed-angle screw and plate system in the treatment of patients with intracapsular fractures of the proximal femur.

### The need for a trial

A review [4] from The Cochrane Database for Systematic Reviews 2007 states:

"Fractures of the thigh bone (femur) near the hip joint (termed intracapsular) may be treated by fixing the fracture (with screws or pins), or alternatively replacing the top of the femur at the hip joint (femoral head) with an artificial hip joint (arthroplasty). This review found that each treatment has its own specific complications. Realigning the bones and fixing the fracture (reduction and internal fixation) is a shorter operation with less blood loss, but is more likely to need a second operation (36% versus 11%). The reason for this is mainly from a failure of the bone to heal in those cases treated with fixation. Internal fixation is associated with less initial operative trauma but has an increased risk of re-operation on the hip."

A search of the national and international clinical trials databases has revealed that there is only one other trial that is being carried out in the USA [7]. This is a commercial trial assessing the use of bone morphogenetic protein (BMP) only. A commercial trial in Leeds, UK investigating the effect of BMP in proximal femoral fractures has recently been abandoned. Otherwise there is no high quality clinical research in this field.

### Good Clinical Practice

The trial will be carried out in accordance with Good Clinical Practice (GCP) and in accordance with the following protocol.

### CONSORT recommendations

The trial will be reported in line with the CONSORT statement [8].

## Methods

### Trial Design

#### Design summary

This trial will be a three arm single centre, standard-of-care controlled, double blinded, pragmatic, randomised clinical trial.

The study will include a standard two-way comparison between PRP and standard-of-care fixation versus standard-of-care fixation alone. This comparison will be the only hypothesis-testing analysis. In addition there will be a subsidiary pilot arm testing fixed-angle screw and plate fixation. This comparison will be a hypothesis-generating analysis only.

The trial is expected to last a total of two years. It is expected that participant recruitment will take one year and final follow-up will be at one year.

The trial was given ethical approval by the Coventry Research Ethics Committee on 6 May 2009.

#### Objectives of the trial

The objectives of this trial are to:

1. test the hypothesis that PRP leads to a reduced incidence of failure of fixation.

2. explore the size of any treatment effect of a novel fixed-angle screw and plate fixation system

#### Measures of efficacy

##### Primary

• The proportion of participants undergoing re-operation for failure of fixation within one year of sustaining the fracture.

##### Secondary

• Radiographic non-union rate at 12 months. Non-union will be defined as "failure of the fracture to show signs of bony union on the anteroposterior or lateral radiograph 1 year after surgery" [9].

• Radiographic evidence of failure of fixation at 6, 12 and 52 weeks

• Radiographic evidence of avascular necrosis at one year

• Magnetic resonance imaging at 6, 12 and 52 weeks. This measure will only be recorded for those participants with capacity.

• The EQ-5D score at 6, 12 and 52 weeks

• Length of index hospital stay

#### Measures of harm and adverse events

##### Expected Adverse Events

• Wound infection

• Venous thrombo-embolic phenomena

• Death

• Pneumonia

• Urinary tract infection

• Blood transfusion

• Failure of fixation

• Cerebrovascular accident

• Acute coronary syndrome

• Myocardial infarction

• Deep vein thrombosis

#### Power and sample size

The minimum clinically important treatment effect of PRP was agreed in discussion with several expert orthopaedic trauma surgeons. Although the figures varied by surgeon, all agreed that an absolute reduction of 15% in fixation failure would be clinically important. The overall rate of fixation failure of all intracapsular fractures of the femur is reported to be 20-35% [10]. Table 1 shows the total sample size with two-sided significance set at 0.05 for various scenarios of minimum clinically relevant difference. Sample sizes were determined using the PS power and sample size software [11].

**Table 1:** Sample sizes calculated for various scenarios

Rate of failure in control group (%)	Rate of failure in the intervention group (%)					
	
	10		15		25	
	
	80% power	90% power	80% power	90% power	80% power	90% power
25	100	133				

30	62	82	121	161		

35	43	57	73	97	329	440

The mortality of patients with intracapsular fractures of the proximal femur is approximately 20% during the first year and this needs to be taken into account in the sample calculation. A recruitment target of 200 participants provides a good margin for unanticipated recruitment problems and loss to follow-up.

In the absence of an agreed method to determine the sample size for a pilot study a group of expert orthopaedic surgeons were consulted. All agreed that a sample of 25 participants in the fixed-angle screw and plate group would be sufficient to provide adequate pilot data.

From a recent audit carried out in our department we know that approximately 450 fractures of the proximal femur are treated operatively per year at University Hospital Coventry and Warwickshire. Approximately 250 of these patients would be eligible for inclusion into this trial. Therefore, even accounting for significant loss to follow-up, the trial sample can be recruited in one year.

#### Eligibility

##### Inclusion criteria

In order that the results of this randomised clinical trial can be generalised as widely as possible, we propose to include all patients, including those with cognitive impairment, admitted with an intracapsular (displaced or undisplaced) fracture of the proximal femur. This pragmatic approach will mean that any conclusions derived will be widely applicable to clinical practice.

##### Exclusion criteria

• All patients who present late following their injury i.e. more than 48 hours after the index fracture.

• Patients with other serious injuries to either lower limb that would interfere with rehabilitation of the index fracture.

• Patients who are managed non-operatively

#### Post-randomisation withdrawals and exclusions

Participants may withdraw from the trial treatment and/or the whole trial at any time without prejudice. If a participant withdraws from the trial treatment he will be followed-up wherever possible and data collected until the end of the trial.

The General Practitioners of those participants who are "lost-to-follow-up" will be contacted in order to attempt to complete the follow-up. Failing this then the Hip Fracture Register will be consulted in order to try to establish up-to-date participant contact details. Participants may be withdrawn from the trial at the discretion of the Chief Investigator due to safety concerns.

#### Consent

An informed consent discussion will be conducted with potential participants after eligibility checks have been performed but prior to randomisation.

Potential participants will be informed about the nature of the trial by the investigator or persons designated by the investigator. This will involve a discussion of purpose and requirements of the trial and the issuing of the participant information sheet.

Patients will be allowed, where possible, at least twenty four hours to consider the information given them prior to being asked to give informed consent to participate in the trial. This period of time will not be allowed to delay any normal standard of care treatment.

Responsibility for recording and dating both verbal and written, signed informed consent will be with the investigator, or persons designated by the investigator, who conducted the informed consent discussion. The following information will be discussed during the consent discussion:

• Benefits of internal fixation of intracapsular proximal femoral fractures

• Risks of internal fixation of intracapsular proximal femoral fractures

• Impact of allocation to different treatment arms of the trial

• Requirements of follow-up

• Benefits of taking part in the trial

For those patients who lack the capacity to give informed consent reasonable efforts will be made to identify a Personal Consultee as described in the *Mental Capacity Act 2005*. If no personal consultee can be identified then a Nominated Consultee will be nominated to advise the research team. The following persons will be approached in the order given in the list below:

i. The patient's General Practitioner

ii. Mr Wade FRCS(Tr&Orth), Consultant Orthopaedic Surgeon UHCW.

At all times the Chief Investigator will act in accordance with the patients' best interests.

#### Recruitment

Participant recruitment will begin in August 2009 and be completed by August 2010. Pre-randomisation eligibility checks will be carried out to ensure that participants are not randomised in error, and informed written consent will be obtained prior to randomisation. Confirmation of these checks will be carried out by the investigator, or persons designated by the investigator, prior to randomisation. Inclusion of the patient in the trial will be flagged on their clinical notes by means of a trial sticker.

#### Treatment allocation

##### Sequence generation

The allocation sequence will be generated randomly. The randomisation will be weighted such that at the end of the trial there will be 25 participants in the fixed-angle screw and plate group and 100 participants in each of the remaining groups. Randomisation will be stratified by displacement of the fracture. Fractures will be defined as undisplaced (Garden grade I or II) or displaced (Garden grade III or IV); Garden's classification of intracapsular fractures is well recognised and universal and it has been validated to distinguish between grades I and II compared with III and IV [12,13]. The surgery will be performed by any of the 16 Consultant Surgeons, two Associate Specialists and 14 Trainees at the University Hospital Coventry and Warwickshire. The large number of surgeons and the wide skill mix should eliminate the 'surgeon effect' such that stratification by surgeon is not required.

##### Allocation concealment

The allocation sequence will be generated using secure, online randomisation via a distant computer generated system administered by The University of York.

##### Allocation implementation

Participants will be enrolled by the trial research associates, co-ordinated by Mr Xavier Griffin. Participants will be assigned to their treatment allocation at the time of surgery by accessing the online randomisation programme. This will allow for treatment allocation to be implemented outside of working hours.

#### Blinding

Participants will be blinded to the treatment allocation. The operating surgeon will not be blinded to the allocation. All outcomes will be assessed by blinded assessors. The primary outcome measure will be determined by the clinical decision of the responsible consultant orthopaedic surgeon who is independent from the trial. The responsible consultant surgeon will not be the operating surgeon in order to maintain the blind. The EQ-5D is a patient reported measure. Patients will be kept blinded until the completion of the trial when the blind is broken. Radiographic outcomes will be assessed by an independent consultant radiologist who is blinded to the treatment allocation. There will be no formal analysis of the success of the blinding.

#### Trial treatments

All participants will have a closed reduction of their fracture. The lower limb will be supported on a fracture table. Internal fixation of the fracture will be achieved through a standard lateral approach with perioperative antibiotic cover in accordance with hospital protocol. Post-operative care will include early active mobilisation managed by a standard physiotherapy rehabilitation regime. All participants will have routine prophylaxis against deep vein thrombosis. Participants will be randomised to one of three groups:

1. Fixed-angle screw and plate fixation

2. Standard of care fixation and placebo injection

3. Standard of care fixation and PRP injection

#### Group 1: Fixed-angle screw and plate fixation

Fixation will be with the Targon FN Head Preserving System as described in the manufacturer's operative technique manual.

#### Group 2: Standard of care fixation

Fixation will be with three parallel cannulated screws. The exact configuration will be left to the discretion of the operating surgeon to ensure the results can be easily generalised. Fixation will be achieved using the standard operative technique.

#### Group 3: Standard of care fixation and PRP injection

Fixation will be with three parallel cannulated screws. The exact configuration will be left to the discretion of the operating surgeon to ensure the results can be easily generalised. Each screw will be advanced up to but not beyond the fracture such that no compression is achieved before the test substance is injected. The guidewire of one screw will then be removed and 5ml of PRP will be injected down the cannulated screw directly into the fracture site under image intensifier guidance. The guidewire will be immediately replaced and the screw/s will then be advanced to compress the fracture site.

#### Concomitant illnesses and medication

Concomitant illnesses and medication will be recorded at trial entry. Changes to these will be recorded at follow-up visits.

#### Interventions and assessments

Table 2 details the assessments and interventions that will be carried out during the period that each participant is involved in the trial.

**Table 2:** Trial assessments and interventions

Serial	Intervention/Measurement	Time (weeks)
1	Operation	0
	Peri-operative complications	

2	AP & lateral radiographs	6
	MRI (subset of sample)	
	Clinical interview	

3	AP & lateral radiographs	12
	MRI (subset of sample)	
	Clinical interview	

4	AP & lateral radiographs	52
	MRI (subset of sample)	
	Clinical interview	

#### End of the trial

The trial will be closed when all participants have completed the one year follow-up visits. Once the trial is completed participants will be treated as per the standard of care.

#### Trial Flow diagram

See figure 1.

**Figure 1:**
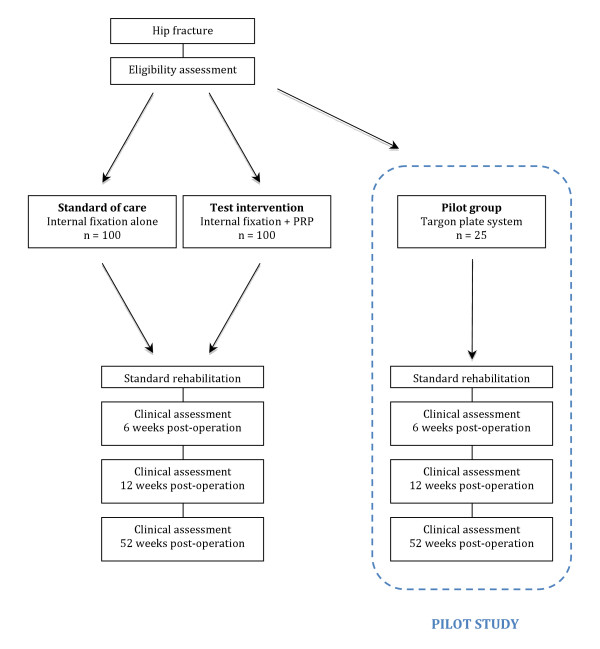
**Trial flow diagram**.

### Data management

#### Database and data management

Data to be collected from participants can be found at table 3. These data will be entered in the trial database. The trial database will be set up by the computer programmer and all specifications agreed between the computer programmer, statistician and trial co-ordinator. The procedure for data entry will be decided when the database is constructed. If electronic databases are required on computers external to the clinical trials unit, they will be compatible with the systems on site and backed-up accordingly. In the case of any interim analysis the database will be frozen at the analysis time point. Data collected after this point will not be included in the interim report.

**Table 3:** Data to be collected during the trial

Serial	Intervention/Measurement	Time (weeks)
1	Peri-operative complications	0

2	EQ-5D score	6
	Radiographic union and fixation	
	Re-operation	
	Readmission	

3	EQ-5D score	12
	Radiographic union and fixation	
	Re-operation	
	Readmission	

4	EQ-5D score	52
	Radiographic union and fixation	
	Avascular necrosis	
	Re-operation	
	Readmission	

The case report forms will be designed by the Trial Co-ordinator in consultation with the Chief Investigator and statistician.

In the event of missing data the relevant clinical databases and case report forms will be accessed to complete the database.

#### Data access and quality assurance

All data collected will be anonymised after the collection of the baseline demographic data for each participant. Identifiable participant data will be held on a separate database and coded with a trial participant code to tag identifiable data to the outcome data.

All data will be stored in a designated storage facility in the Clinical Sciences Building on the research site at the University of Warwick. Data will be stored on password protected university computers in a restricted access building.

#### Archiving of trial data

Data will be archived in accordance with The University of Warwick clinical trials unit guidance.

#### Data monitoring committee

There will be a data monitoring committee convened for this trial.

### Statistical analysis plan

#### Analysis of efficacy

##### PRP vs standard-of-care (parallel cannulated screws)

The primary outcome measure, the proportion of patients requiring re-operation for failure of fixation within one year of sustaining the fracture, will be analysed using a chi-squared test for differences between internal fixation alone (control) and internal fixation and PRP (PRP) on an intention-to-treat basis. Treatments will be considered to differ significantly if p-values are <0.05 (5% level). Similarly, chi-squared tests will be used to assess the significance of observed differences for the secondary proportional outcome measures. If the numbers in the contingency tables are small (cells with values below 10) then Fisher's exact test will be used in preference to the chi-squared test. In addition to the main analysis, that will report treatment group effects for the primary outcome measure, a subsidiary analysis will use a multiple linear regression model to investigate the relationship between each patient's EQ-5D Score at 12 months and the treatment arm, age, gender, dementia and fracture displacement for each patient. Estimates, and 95% confidence intervals, from the regression model, and unadjusted results from t-tests will be reported and inferences made on the significance of the treatment effect. All analyses will be based upon an intention-to-treat analysis so missing data due to protocol violations will not be relevant. The primary outcome measure in this study has been chosen in order to limit the possibility of losing data from failed participant follow-up. The primary measure can be sourced from the patient, relative, GP or national hp fracture database.

##### Fixed-angle plate and screws vs standard-of-care (parallel cannulated screws)

No formal inference statistical analysis will be conducted on the data from the pilot arm of the study. The proportional primary event rate, mean estimates and variability of the secondary measures in the two groups will be described. Additionally an estimate of the size of the treatment effect due to the fixed-angle plate will be made to inform further study designs.

#### Subgroup analyses

Planned subgroup analyses will be undertaken only for fracture displacement (displaced vs undisplaced), dementia and appropriate age groups.

#### Analysis of adverse events

The number and temporal pattern of adverse events will be investigated to assess if these differ between treatment groups.

### Trial organisation and oversight

#### Trial steering committee

A trial steering committee will be convened and independently chaired in accordance with the University of Warwick Clinical Trials Unit standard operating procedures. In addition to the independent chair, Mr M Costa, Mr X Griffin, Dr J Achten and Dr N Parsons will form the committee. All issues pertaining to the management of the trial will be co-ordinated by the trial steering committee. The schedule for meetings of the committee will be as follows:

Meeting 1: Trial commencement

Meeting 2: Interim meeting at 50% recruitment

Subsequent meetings: End of trial

#### Data monitoring committee

A data monitoring committee will be convened once the trial is 50% recruited. The committee will be chaired by Mr S Drew, University Hospital Coventry and Warwickshire NHS Trust.

#### Trial registration

The trial is registered with the Current Controlled Trials register ISRCTN49197425. The trial has been adopted by the National Institute for Health Research Clinical Research Network Portfolio NIHR CRN Study ID: 7762.

#### Project timetable and milestones

Trial recruitment commenced August 2009

All participants recruited August 2010

Trial completed August 2011

Trial reported December 2011

#### Unblinding

The blind will only be broken for clinical management purposes. In exceptional circumstances beyond this agreement will be sought from the Chief Investigator and statistician before the blind is broken.

#### Interim analysis

There will be no formal interim analysis conducted.

#### Indemnity/compensation/insurance

All issues of indemnity, compensation and insurance are detailed in the joint sponsorship agreement between the University of Warwick and University Hospital Coventry and Warwickshire NHS Trust.

#### Essential documents

All essential documentation will be stored as specified under the guidance from the clinical trials unit.

#### Monitoring and quality assurance policy

The Chief Investigator and data entry technician will conduct sampling of the database quarterly in order to identify any problems in trial procedures.

#### Dissemination and publication

The results of this trial will be disseminated to the trauma and orthopaedic surgery community via presentations at national and international meetings as well as publication in peer reviewed journals.

#### Financial support

The trial will be funded by the Furlong Research Charitable Foundation and the Bupa Foundation.

## Competing interests

Gian Medical have agreed to provide the consumables for the production of the platelet-rich plasma used in the treatment of some participants in this trial.

BBraun have agreed to provide the Targon FN Head Preserving System used in the treatment of some participants in this trial.

Neither Gian Medical nor BBraun have any rights to the intellectual property generated from the data produced by this trial.

XG is funded by the Furlong Research Charitable Foundation to carry out this research.

The trial is funded by a grant from the Bupa Foundation.

## Authors' contributions

XG and MC developed the trial concept and design. All authors made significant contributions to the design, drafting and critical revision of the trial protocol. XG will co-ordinate participant recruitment and follow-up. All authors will be responsible for data interpretation and reporting of the trial. All authors read and approved the final manuscript.

## Pre-publication history

The pre-publication history for this paper can be accessed here:

http://www.biomedcentral.com/1471-2474/11/184/prepub
